# The unseen world: reflections on Leeuwenhoek (1677) ‘Concerning little animals’

**DOI:** 10.1098/rstb.2014.0344

**Published:** 2015-04-19

**Authors:** Nick Lane

**Affiliations:** Department of Genetics, Evolution and Environment, University College London, London WC1E 6BT, UK

**Keywords:** Leeuwenhoek, animalcule, protozoa, bacteria, eukaryote, tree of life

## Abstract

Leeuwenhoek's 1677 paper, the famous ‘letter on the protozoa’, gives the first detailed description of protists and bacteria living in a range of environments. The colloquial, diaristic style conceals the workings of a startlingly original experimental mind. Later scientists could not match the resolution and clarity of Leeuwenhoek's microscopes, so his discoveries were doubted or even dismissed over the following centuries, limiting their direct influence on the history of biology; but work in the twentieth century confirmed Leeuwenhoek's discovery of bacterial cells, with a resolution of less than 1 µm. Leeuwenhoek delighted most in the forms, interactions and behaviour of his little ‘animalcules', which inhabited a previously unimagined microcosmos. In these reflections on the scientific reach of Leeuwenhoek's ideas and observations, I equate his questions with the preoccupations of our genomic era: what is the nature of Leeuwenhoek's animalcules, where do they come from, how do they relate to each other? Even with the powerful tools of modern biology, the answers are far from resolved—these questions still challenge our understanding of microbial evolution. This commentary was written to celebrate the 350th anniversary of the journal *Philosophical Transactions* *of the Royal Society*.

My work, which I've done for a long time, was not pursued in order to gain the praise I now enjoy, but chiefly from a craving after knowledge, which I notice resides in me more than most other men. Leeuwenhoek, Letter of 12 June 1716

Leeuwenhoek is universally acknowledged as the father of microbiology. He discovered both protists and bacteria [1]. More than being the first to see this unimagined world of ‘animalcules', he was the first even to think of looking—certainly, the first with the power to see. Using his own deceptively simple, single-lensed microscopes, he did not merely observe, but conducted ingenious experiments, exploring and manipulating his microscopic universe with a curiosity that belied his lack of a map or bearings. Leeuwenhoek (figure 1) was a pioneer, a scientist of the highest calibre, yet his reputation suffered at the hands of those who envied his fame or scorned his unschooled origins, as well as through his own mistrustful secrecy of his methods, which opened a world that others could not comprehend. The verification of this new world by the natural philosophers of the nascent Royal Society laid out the ground rules that still delineate science today, but the freshness and wonder, the sheer thrill of Leeuwenhoek's discoveries, transmit directly down the centuries to biologists today. Microbiologists and phylogeneticists continue to argue about the nature of Leeuwenhoek's little animals, if in more elaborate terms. Only now are we beginning to find answers—and surprisingly uncertain answers—to the questions that drove Leeuwenhoek: where did this multitude of tiny ‘animals' come from, why such variety in size and behaviour; how to distinguish and classify them?

**Figure 1.**
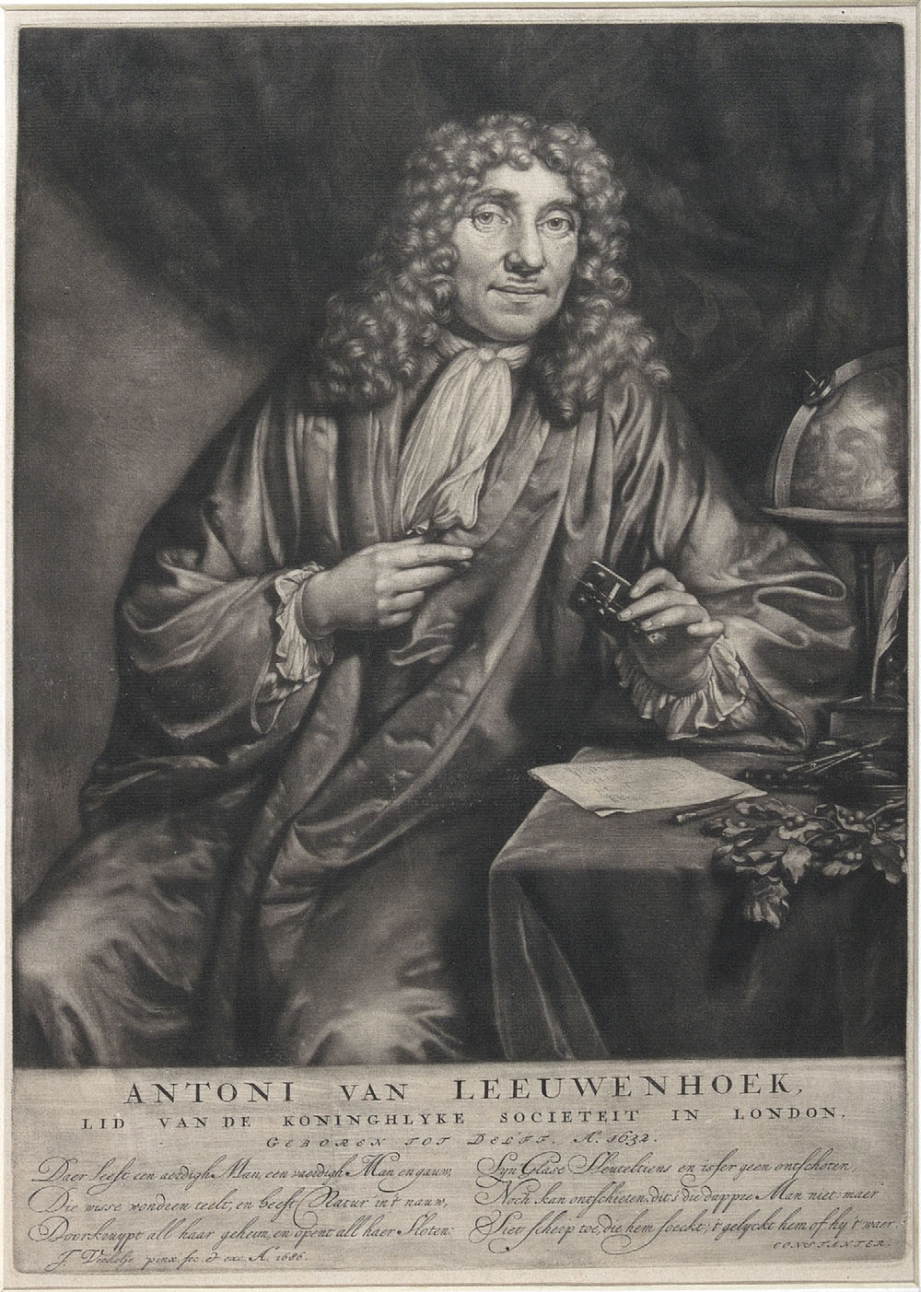
Portrait of Leeuwenhoek by Jan Verkolje, 1686, at age 54. Copyright © The Royal Society.

Leeuwenhoek's 1677 paper [1] was not his first contribution to *Philosophical Transactions*, nor was it his first mention of little animals living in water. The paper was translated (‘English'd’) from low Dutch, and excerpted to half its original length by the redoubtable Henry Oldenburg, first Secretary of the Royal Society and founding editor of *Philosophical Transactions*. Oldenburg corresponded so widely across Europe that he was imprisoned in the Tower for suspected espionage in 1667, during the Second Anglo-Dutch War (when the Dutch colony of New Amsterdam was renamed New York). Oldenburg later adopted a pseudonym, the anagram ‘Grubendol’, to avert suspicion (it would arouse mine). Among his regular Dutch correspondents were the surgeon Regnerus de Graaf and statesman Constantijn Huygens, father of famed astronomer Christiaan Huygens, both of whom wrote epistles to Oldenburg introducing ‘the exceedingly curious and industrious' Leeuwenhoek, Huygens adding the helpful note ‘or Leawenhook, according to your orthographie’ [2]. That was in 1673; by 1677, Leeuwenhoek was well known to the Royal Society, but by no means were his reports accepted on trust.

Oldenburg published several of Leeuwenhoek's letters in 1673 and 1674, which dealt with interesting but uncontentious matters, such as the structure of the bee sting. Equivalent microscopic structures of objects visible to the naked eye had been illuminated by Robert Hooke in his *Micrographia* nearly a decade earlier; indeed, it is to Hooke that we owe the word ‘cell’, which he used to denote the boxy spaces (reminiscent of the small rooms in a monastery) that make up the structure of cork [3]. From some of Leeuwenhoek's slightly waspish remarks in his early letters, he had almost certainly seen a copy of *Micrographia* on his visit to London in 1667 or 1668, when the book was practically a fashion accessory (‘the most ingenious book I read in all my life’, wrote Pepys, who stayed up all night with it; Pepys reputedly stayed up all night often, though rarely with a book). Leeuwenhoek first courted controversy in a letter of September 1674. Describing a nearby lake, Berkelse Mere, he noted that its water was very clear in winter ‘but at the beginning or middle of summer it becomes whitish, and there are then little green clouds floating in it’ [4]. These clouds contained wispy ‘green streaks, spirally wound serpent-wise, and orderly arranged’—the beautiful green alga *Spirogyra*. Then came Leeuwenhoek's first mention of little animals: ‘among these streaks there were besides very many little animalcules … And the motion of most of these animalcules in the water was so swift, and so various upwards, downwards and round about that ‘twas wonderful to see: and I judged that some of these little creatures were above a thousand times smaller than the smallest ones I have ever yet seen upon the rind of cheese’ (by which he meant mites) [4].

Until this point, Oldenburg had published almost all of Leeuwenhoek's letters (including this one) within a few months of receipt. Now, he drew pause. Of the next 12 letters sent by Leeuwenhoek, only three were published, and none that touched on animalcules. Oldenburg had every reason to be suspicious; as Leeuwenhoek wrote to Hooke a few years later ‘I suffer many contradictions and oft-times hear it said that I do but tell fairy tales about the little animals' [5]. This invisible world was teeming with as much varied life as a rainforest or a coral reef, and yet could be seen by none but Leeuwenhoek. No wonder Oldenburg and his colleagues had doubts. Set against this background, Leeuwenhoek wrote his eighteenth letter to the Royal Society, dated October 1676, the celebrated ‘letter on the protozoa’, which Oldenburg excerpted, translated and published as the 1677 paper. It opens with a bang: ‘In 1675 I discovered living creatures in Rain water which had stood but few days in a new earthen pot, glased blew [i.e. painted blue] within. This invited me to view this water with great attention, especially those little animals appearing to me ten thousand times less than those represented by Mons. Swamerdam and called by him *Water fleas* or *Water-lice*, which may be perceived in the water with the naked eye’ [1].

Clifford Dobell, in his delightful biography published in 1932 (300 years after Leeuwenhoek's birth), notes that Oldenburg's translation is good but not perfect [6]. That's not surprising. While Oldenburg knew the language, he had no knowledge of the organisms themselves. In contrast, Dobell was a distinguished microbiologist, a Fellow of the Royal Society, and had the great benefit of hindsight. His biography was a labour of love, written over 25 years, frequently in the middle of the night, while carrying out his own research on intestinal protozoa and other protists. Dobell taught himself Dutch and translated Leeuwenhoek's letters painstakingly—written, as they were, in a colloquial Dutch no longer in use, and in the beautiful but scarcely legible hand of a copyist (figure 2). Dobell revelled in the precise beauty of Leeuwenhoek's descriptions of *Euglena*, *Vorticella* and many other protists and bacteria, which leapt off the page, immediately recognizable to this expert kindred spirit. Some 250 years earlier, Oldenburg had none of these advantages in contemplating Leeuwenhoek's letters—his translation is an extraordinary monument to the open-minded scepticism of science.

**Figure 2.**
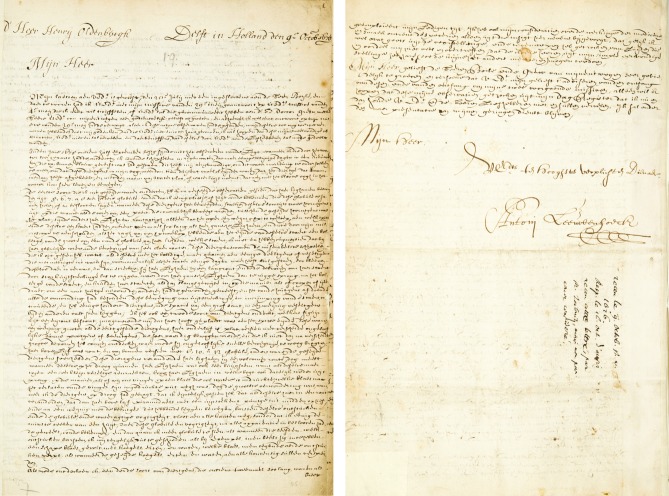
First and last pages of Leeuwenhoek's 1676 letter to Oldenburg, in the hand of a copyist. Copyright © The Royal Society.

I sketch this background because the paper itself is unusual even in Leeuwenhoek's oeuvre, taking the form of a diary. On a cursory reading, it seems almost embarrassingly naive to the modern ear—the earthen pot ‘glased blew within’ in the first sentence is a good example (but see [7] for a discussion of Dutch prose style in the seventeenth century). We learn that on ‘the 17th of this month of June it rained very hard; and I catched some of that rain water in a new Porcelain dish, which had never been used before, but found no living creatures at all in it’ [1]. On it goes, with precise but apparently irrelevant details. ‘In the open Court of my house I have a well, which is about 15 foot deep, before one comes to the water. It is encompassed with high walls, so that the Sun, though in Cancer, yet can hardly shine much upon it. This water comes out of the ground, which is sandy, with such a power, that when I have laboured to empty the well, I could not so do it but there remained ever a foots depth of water in it. This water is in Summer time so cold, that you cannot possibly endure your hand in it for any reasonable time’ [1]. And my favourite: ‘July 27 1676. I went to the sea-side, at Schlevelingen, the wind coming from the Sea with a very warm Sun-shine; and viewing some of the Sea-water very attentively, I discovered divers living animals therein. I gave to a man, that went into the Sea to wash himself, a new glass-bottle, bought on purpose for that end, intreating him, that being on the Sea, he would first wash it well twice, or thrice, and then fill it full of the Sea-water; which desire of mine having been complied with, I tyed the bottle close with a clean bladder’ [1].

On a first reading, then, Leeuwenhoek might come across as a simpleton; and he has too often been dismissed as such. One can only smile at the image of Leeuwenhoek on the beach, pressing his pre-prepared bottles onto strangers. But which details are important? How should he have charted this abundant new world? We need to appreciate several points. This letter was intended to defend his discoveries—‘merely so as to make my observations more credible in England and elsewhere’ [8]. Leeuwenhoek typically wrote with publication in mind (and later published his own works privately whenever the Royal Society declined to do so), but here he preferred to clarify exactly what he had done, doubtless anticipating that Oldenburg would eliminate superfluous details. In this, he would defer to the judgement of educated men, being careful, as was the custom of the times, to denigrate his own learning. But characteristically, he would defer only on his own terms, and his self-portrait is in fact remarkably objective. ‘I have oft-times been besought, by divers gentlemen, to set down on paper what I have beheld through my newly invented *Microscopia*: but I have generally declined; first, because I have no style, or pen, wherewith to express my thoughts properly; second, because I have not been brought up to languages or arts, but only to business; and in the third place, because I do not gladly suffer contradiction or censure from others' [9]. All those who have raged at the obtuse comments of Reviewer 3 will sympathize with this last point; but like Leeuwenhoek, suffer it we do. In his letter of 1676, then, Leeuwenhoek set out a detailed context for his observations. Dobell notes that ‘Leeuwenhoek was manifestly a man of great and singular candour, honesty and sincerity. He was religiously plain and straightforward in all he did, and therefore sometimes almost immodestly frank in describing his observations. It never occurred to him that Truth could appear indecent’ [10].

On a closer reading, the colloquial manner of Leeuwenhoek's letter conceals the workings of his precise and methodical mind. Leeuwenhoek was acutely aware of contamination; he replenished evaporated water with snow-water, the purest then available, making every effort not to introduce little animals from any other source. He sampled water from many different sources—his well, the sea, rain water, drain pipes, lakes—always taking care to clean his receptacles. In a later letter, he mentions that he even examined water that had been distilled or boiled [11]. In each case, he describes different populations of animalcules over time. Time is critical. Frequently, he observes nothing for a week, checking each day, before reporting a profusion of little animals of diverse types, replicating themselves over several days before dying back again. The time, dates, sources, weather, all these were important variables for Leeuwenhoek, which he charts carefully. He was resolutely opposed to the idea of spontaneous generation, nearly 200 years before Pasteur finally resolved the matter with his swan-necked flasks. Leeuwenhoek later described the procreation of cells via copulation or schism to release daughter cells in arresting detail. But his early disbelief of spontaneous generation is implicit in the comparisons of his 1677 paper, in his care to avoid contamination, and his estimation of rates of growth.

Leeuwenhoek also reports experiments, adding peppercorns to water, both crushed and uncrushed (as well as ginger, cloves, nutmeg and vinegar, omitted from Oldenburg's excerpts for *Philosophical Transactions*). In these infusions, Leeuwenhoek observed an astonishing proliferation of tiny animals ‘incredibly small; nay, so small, in my sight, that I judged that even if 100 of these very wee animals lay stretched out one against another, they could not reach the length of a grain of course sand; and if this be true, then ten hundred thousand of these living creatures could scarce equal the bulk of a course grain of sand’ [1]. Again, the colloquial language deceives. In a clarification sent to Constantijn Huygens and Hooke, Leeuwenhoek writes ‘Let's assume that such a sand-grain is so big, that 80 of them, lying one against the other, would make up the length of one inch’ [12]. He goes on to calculate the number of animalcules in a cubic inch; for our purposes here, his calculation puts the length of his ‘very wee animals' at less than 3 µm. Bacteria. (He later describes bacterial motility unequivocally [13]). He also notes that he deliberately underestimates the number of bacteria in a drop of water—‘for the reason that the number of animalcules in so small a quantity of water would else be so big, that ‘twould not be credited: and when I stated in my letter of 9th October 1676, that there were upwards of 1 000 000 living creatures in one drop of pepper-water, I might with truth have put the number at eight times as many’ [14]. An innocent, early example of spinning data to sell to a journal?

But the natural philosophers of the Royal Society, in pioneering the methods we still use in science today, were not easily spun. Leeuwenhoek's letter had been read aloud over several sessions and attracted great interest, verging on consternation. Oldenburg wrote to Leeuwenhoek, asking him to ‘acquaint us with his method of observing, that others may confirm such Observations as these’, and to provide drawings [15]. Leeuwenhoek declined, throughout his life, to give any description of his microscopical methods (‘for reasons best known to himself’, said Hooke; though science has hardly resolved the issue of intellectual property since then). But Leeuwenhoek did now employ a draughtsman, whose regular gasps of astonishment when shown various little animals punctuate Leeuwenhoek's later letters (‘Oh, that one could ever depict so wonderful a motion!’). Some of these limner's drawings are shown in figure 3. Leeuwenhoek also sent eight testimonies from gentlemen of repute—a Lutheran minister, a notary and a barrister, among others. It is striking to the modern reader that none of these gentlemen were natural philosophers acquainted with the methods of science; but according to the historian Steven Shapin, it was the bond of the gentleman that counted. The practice of signed testimonies from gentlemen was common in the seventeenth century; the fact that Leeuwenhoek called upon eight such testimonies attests to the unprecedented character of his findings, but also perhaps to his lower social standing [18].

**Figure 3.**
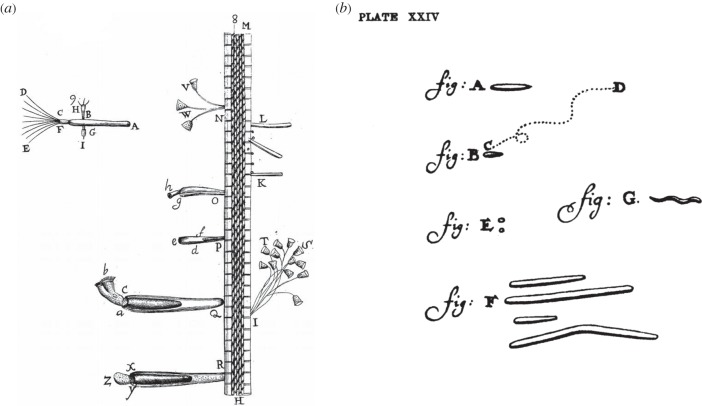
(*a*) Rotifers, hydra and vorticellids associated with a duckweed root, from a Delft canal. From Leeuwenhoek [16]. (*b*) Bacteria from Leeuwenhoek's mouth; the dotted line portrays movement. From Leeuwenhoek [17]. Copyright © The Royal Society.

No doubt all this was helpful, but it was countered by letters from others such as Christiaan Huygens (son of Constanijn), then in Paris, who at that time remained sceptical, as was his wont: ‘I should greatly like to know how much credence our Mr Leeuwenhoek's observations obtain among you. He resolves everything into little globules; but for my part, after vainly trying to see some of the things which he sees, I much misdoubt me whether they be not illusions of his sight’ [19]. The Royal Society tasked Nehemiah Grew, the botanist, to reproduce Leeuwenhoek's work, but Grew failed; so in 1677, on succeeding Grew as Secretary, Hooke himself turned his mind back to microscopy. Hooke too initially failed, but on his third attempt to reproduce Leeuwenhoek's findings with pepper-water (and other infusions), Hooke did succeed in seeing the animalcules—‘some of these so exceeding small that millions of millions might be contained in one drop of water’ [20] (actually far less precise than Leeuwenhoek). He went on to write ‘It seems very wonderful that there should be such an infinite number of animalls in soe imperceptible quantity of matter. That these animalls should be soe perfectly shaped and indeed with such curious organs of motion as to be able to move nimbly, to turne, stay, accelerate and retard their progresse at pleasure. And it was not less surprising to find that these were gygantick monsters [protozoa] in comparison of a lesser sort which almost filled the water [bacteria]’ [21].

Unlike Leeuwenhoek, Hooke gave precise details of his microscopical methods, and demonstrated them before the gathered fellows, including Sir Christopher Wren, later publishing both his methods and observations in *Microscopium* (1678) [20]. He even taught himself Dutch, so that he could read the letters of the ‘ingenious Mr Leeuwenhoek’. As noted by the microscopist Brian J. Ford [22] and microbiologist Howard Gest [23], Hooke was a central and too-often overlooked figure in the history of microbiology: his earlier book *Micrographia* (1665) most likely inspired Leeuwenhoek to begin his own microscopical studies. Without Hooke's support and verification—a task beyond several of the best microscopists of the age, including Grew—Leeuwenhoek might easily have been dismissed as a charlatan. Instead, through Hooke's impressive demonstrations, and with the direct support of the patron of the Royal Society, King Charles II, Leeuwenhoek was elected a Fellow in 1680. Others had independently changed their view of Leeuwenhoek in the interim, but that did little to alter the course of events. Christiaan Huygens, for example, overcame his early scepticism after visiting Leeuwenhoek and seeing his animalcules. He went on to grind his own lenses, observing various protists himself [24]. Indeed, Huygens made a number of pioneering observations, but these remained in manuscript and were unpublished until the turn of the twentieth century [25].

Ironically, Hooke's admirable comments on the construction of microscopes might have undermined Leeuwenhoek's later reputation. Hooke made various types of microscope. He much preferred using larger instruments with two lenses, but in the Preface to *Micrographia* [26] he also described how to make ‘simple’ microscopes with a single lens—what became known as a Leeuwenhoek microscope [27]. The lens is produced by melting Venice glass into thin threads, containing little globules, which are then ground and polished, and mounted against a needle hole pricked through a thin plate of brass (figure 4). ‘If … an Object, plac'd very near, be look'd at through it, it will both magnifie and make some objects more distinct than any of the great Microscopes. But because these, though exceeding easily made, are yet very troublesome to be us'd, because of their smallness, and the nearness of the Object; therefore to prevent both of these, and yet have only two refractions, I provided me a Tube of Brass' [26]. In 1678, Hooke reiterated his dislike of single-lens microscopes: ‘I have found the use of them offensive to my eye, and to have much strained the sight, which was the reason why I omitted to make use of them, though in truth they make the object appear much more clear and distinct, and magnifie as much as the double Microscopes: nay to those whose eyes can well endure it, ‘tis possible with a single Microscope to make discoveries much better than with a double one, because the colours which do much disturb the clear vision in double Microscopes is clearly avoided and prevented with the single’ [20].

**Figure 4.**
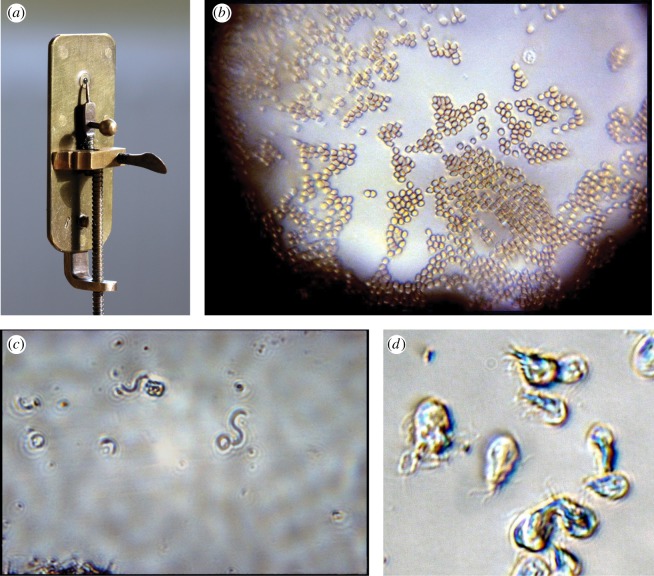
(*a*) Replica of a single-lens microscope by Leeuwenhoek (Image by Jeroen Rouwkema. Licensed under CC BY-SA 3.0 via Wikimedia Commons). (*b,d*) Photomicrographs taken using simple single-lens microscopes including one of Leeuwenhoek's originals in Utrecht, by Brian Ford (Copyright © Brian J. Ford). (*b*) An air-dried smear of Ford's own blood through the original van Leeuwenhoek microscope at Utrecht, showing red blood cells and a granulocyte with its lobed nucleus (upper right; about 2 µm in diameter). (*c*) Spiral bacteria (*Spirillum volutans*) imaged through a replica microscope with a lens ground from spinel; each bacterial cell is about 20 µm in length. (*d*) The intestinal protist parasite *Giardia intestinalis* imaged through a replica soda-glass produced by Brian Ford [28,29].

It seems that Hooke's aversion to simple single-lens microscopes passed on down the generations, but not his appreciation of their merits. The compound microscope, with its refractive aberrations, became the tool of choice, and Leeuwenhoek's microscopes were quietly forgotten, their oblivion hastened by Leeuwenhoek's own secrecy, notwithstanding his gift of 13 microscopes, with corresponding specimens, to the Royal Society on his death in 1723 at the age of 90. Leeuwenhoek had actively discouraged teaching his methods, for reasons that are troubling today in an age when education is open to all. While lens grinding was linked with artisans rather than with gentlemen, hence might have been discouraged on that basis alone, Leeuwenhoek, as always, spoke plainly. In a letter to Leibnitz, he wrote ‘To train young people to grind lenses, and to found a sort of school for this purpose, I can't see there'd be much use: because many students at Leyden have already been fired by my discoveries and my lens grinding … But what's come of it? Nothing, as far as I know: because most students go there to make money out of science, or to get a reputation in the learned world. But in lens grinding, and discovering things hidden from our sight, these count for nought. And I'm satisfied too that not one man in a thousand is capable of such study, because it needs much time, and spending much money; and you must always keep on thinking about these things, if you are to get any results. And over and above all, most men are not curious to know: nay, some even make no bones about saying: What does it matter whether we know this or not?’ [30]. Most scientists, I imagine, would see themselves as that one man in a thousand; it is our task today to persuade others that it does indeed matter, not for any immediate benefit, but for the sake of curiosity and its unknowable contribution to the sum of human knowledge and wellbeing.

The dominant use of compound microscopes over the following centuries meant that the brief blaze of Leeuwenhoek's discoveries was nearly extinguished until the great compound microscope makers of the early-nineteenth century, notably Joseph Bancks (who also produced some high-powered single-lens microscopes, used by Robert Brown in his discovery of Brownian motion and cytoplasmic streaming, and by Darwin aboard the Beagle). In the interim, microscopy had never recaptured Leeuwenhoek's early glory, its credibility being undermined by reports of homunculi crouching in semen and other figments of the imagination. The concept of preformation was called into serious question from the 1740s, beginning with Abraham Trembley's work on the regeneration of freshwater polyps [31]. In the 1750s, Linnaeus scarcely troubled himself with the classification of microbes; he dumped the whole lot into the phylum *Vermes* (‘worms'), genus *Chaos* (formless). The damaging accusation of seeing things that were not there, combined with Linnaeus's insinuated absence of structure, meant that few believed Leeuwenhoek could have seen cells as small as bacteria; even the empathetic Dobell struggled to conceive what magical form of lighting Leeuwenhoek must have employed to view his specimens. Only the galvanizing work of Brian J. Ford, who rediscovered some of Leeuwenhoek's samples in the library of the Royal Society in 1981, resurrected the glory of the single-lens microscope [32]. Ford photographed Leeuwenhoek's original specimens using one of his surviving microscopes in Utrecht, and demonstrated a remarkable resolution of less than 1 µm [33] (figure 4). That left little scope for disbelief: plainly, Leeuwenhoek really did see much of what he claimed.

So what is Leeuwenhoek's legacy? Most of his discoveries were forgotten, and only rediscovered in the nineteenth century, 150 years later, being then interpreted in the context of the newly developing cell theory, with little reference back to Leeuwenhoek himself. In this regard Leeuwenhoek's legacy is analogous to that of Gregor Mendel, likewise rediscovered at a time when others were exploring similar ideas. Leeuwenhoek's work, of course, ranged far beyond microbiology. In all, he sent around 200 letters to the Royal Society, 112 of which were published, touching on many aspects of biology and even mineralogy. He remains the most highly published author in the journal. He is considered to be the founder of many fields, but none of them more important than his astonishing discoveries in microbiology, and none conveyed with such delight. Leeuwenhoek was captivated by his animalcules. ‘Among all the marvels that I have discovered in nature’, he wrote, ‘these are the most marvellous of all’ [34]. His exhilaration in discovery, combined with a fearless and surefooted interpretation of unknown vistas, is for me Leeuwenhoek's true legacy. It is a spirit effervescent in many later pioneers of microbiology, indeed in science more generally. And many of the problems that beset Leeuwenhoek troubled them too.

Take the ultrastructure of cells, especially protists. Leeuwenhoek could clearly see ‘little feet’ (cilia) and also the budding offspring of cells, but he saw much more than that. I'm struck by this passage in the 1677 paper, describing an ‘egg-shaped’ animalcule (which Dobell tentatively identified as the ciliate *Colpidium colpoda* [35]): ‘Their body did consist, within, of 10, 12, or 14 globuls, which lay separate from each other. When I put these *animalcula* in a dry place, they then changed their body into a perfect round, and often burst asunder, & the globuls, together with some aqueous particles, spred themselves every where about, without my being able to discern any other remains. These globuls, which in the bursting of these creatures did flow asunder here and there, were about the bigness of the first very small creatures [bacteria]. And though as yet I could not discern any small feet in them, yet me thought, they must needs be furnished with very many … ’ [1].

While the ‘globuls' in *C. colpoda* were probably mostly food vacuoles, as well as the macronucleus, Leeuwenhoek's comparison with bacteria leaves open the tantalizing possibility that he had even seen organelles such as mitochondria, which with a diameter of 0.5–1 µm would have pushed his microscopical resolution to the limits. Some 250 years later, this equivalence between intracellular ‘globuls' and free-living bacteria was pursued by the early-twentieth century pioneers of endosymbiotic theory, notably the Russian Konstantin Mereschkowski, Frenchman Paul Portier and American Ivan Wallin, the latter pair independently going so far as to argue that mitochondria could be cultivated [36]. The idea of ‘symbiogenesis' was famously ridiculed by the American cell biologist E.B. Wilson, who summed up the prevailing attitude: ‘To many, no doubt, such speculations may appear too fantastic for present mention in polite biological society; nevertheless, it is within the range of possibility that they may some day call for serious consideration’ [37]. Another half-century was to elapse before Lynn Margulis and others demonstrated that mitochondria and chloroplasts do indeed derive from bacterial endosymbionts [38]; and even then, not without a fight. I doubt that the idea of endosymbiosis would have shocked Leeuwenhoek; nor would he have been much surprised by the contemptuous disbelief of many biologists over decades.

Another unifying theory came from biochemistry, and fittingly drew inspiration from Leeuwenhoek's hometown of Delft (described by the Earl of Leicester, once Governor-General of the Netherlands, as ‘another London almost for beauty and fairness'). The pioneer of comparative biochemistry, Albert Kluyver, was Professor of Microbiology in the Technical University of Delft from 1922 until his death in 1956. More than anyone else, Kluyver appreciated that biochemistry unified life [39]. He realized that different types of respiration (he cites sulfate reduction, denitrification and methanogenesis) are fundamentally equivalent, all involving the transfer of electrons from a donor to an acceptor. He appreciated that all forms of respiration and fermentation are united in that they all drive growth by means of phosphorylation. Such parallels made the startling differences between cells explicable, a discovery he cherished as ‘highly edifying to the scientific mind’ [40]. He expressed this unity in the awkward phrase ‘From elephant to butyric acid bacterium—it is all the same’, later paraphrased, more memorably but without attribution, by François Jacob and Jacques Monod as ‘that old axiom ‘what is true for bacteria is also true for elephants’’. Kluyver, in a seminal passage, recognized that the fundamental unity of biochemistry ‘opens the way for a better appreciation of evolutionary developments which have taken place in the microbial world, since the antithesis between the aerobic and anaerobic mode of life has been largely removed’ [40].

The unity of biochemistry, then, gave the first insights into the evolution of the astonishing variety of ‘little animals', which until then had remained a mystery, their provenance as wholly unknown as in Leeuwenhoek's time. Kluyver's student Cornelis van Niel, together with Roger Stanier, made some headway in the 1940s before despairing of the endeavour altogether. By the time they published their famous essay ‘The concept of a bacterium’ in 1961 they no longer cared to defend their own earlier taxonomic systems [41]; they sought only to distinguish bacteria (prokaryotic cells, lacking a nucleus) from larger eukaryotic protists, all of which have a nucleus. In this, they were remarkably perspicacious, commenting: ‘The differences between eukaryotic and prokaryotic cells are not expressed in any gross features of cellular function; they reside rather in *differences with respect to the detailed organization of the cellular machinery*’ [41]. They cite the examples of respiration and photosynthesis, found in both eukaryotic and prokaryotic cells: ‘But in the prokaryotic cell, these metabolic unit processes are performed by an apparatus which always shows a much smaller degree of specific organization. In fact, one can say that *no unit of structure smaller than the cell in its entirety is recognizable as the site of either metabolic unit process*' [41]. This is a beautiful insight, worthy of Leeuwenhoek himself. In eukaryotes, respiration and photosynthesis are conducted in mitochondria and chloroplasts, respectively, and continue perfectly well in isolation from the rest of the cell, as all the soluble enzymes needed are constrained within the bioenergetic membranes of the organelle. In bacteria, by contrast, the enzymes required are split between the cell membrane (whether invaginated or otherwise) and the cytosol, making the bacterium as a whole the indivisible functional unit. This distinction applies as much to cyanobacteria (classed as algae, not bacteria, by Ernst Haeckel and later systematists) as to other bacteria. Stanier and van Niel therefore argued that bacteria are a single (monophyletic) group, all similar in their basic plan, but insisted that any further attempts to define phylogeny were hopeless.

The timing was unfortunate. Francis Crick had already advocated the use of molecular sequences as a wonderfully sensitive phylogenetic signal, writing in 1958: ‘Biologists should realize that before long we shall have a subject which might be called ‘protein taxonomy’—the study of amino acid sequences of proteins of an organism and the comparison of them between species. It can be argued that these sequences are the most delicate expression possible of the phenotype of an organism and that vast amounts of evolutionary information may be hidden away within them’ [42]. Soon afterwards, Zuckerkandl & Pauling [43] formalized the argument with sequence data; and a mere two decades later, Carl Woese published his first tree of life [44]. Woese [45] was soon dismissing Stanier and van Niel as epitomising the dark ages of microbiology, when microbiologists had given up any prospect of a true phylogeny. Woese's tree was based on ribosomal RNA. He showed that prokaryotes are not monophyletic at all, but subdivide into two great domains, the bacteria and archaea. Later work, which used other methods to ‘root’ the tree [46], portrayed the eukaryotes as a ‘sister group’ to the archaea [47]. For the first time, it seemed possible to reconstruct the evolutionary relationships between Leeuwenhoek's animalcules in an evolutionary tree of life. Woese and his co-workers went so far as to argue that the term prokaryote was obsolete, being an invalid negative definition (i.e. prokaryotes are defined by the absence of a nucleus; [48]). The three domains tree is still the standard text book view. Even so, for all its revolutionary appeal, Woese's tree is the apotheosis of a reductionist molecular view of evolution, based on constructing trees from a single gene. It is ironic that, later in life, Woese called for a more holistic biology, while refusing to countenance the limitations of his single-gene tree [49].

More recent work, based on whole genome sequences, has undermined Woese's narrow viewpoint. While the sisterhood of archaea and eukaryotes is upheld for a core of informational genes—genes involved in DNA replication, transcription and translation—it is not at all true for most other genes in eukaryotes, which are more closely related to bacteria than archaea. Woese's iconic tree is therefore profoundly misleading, and should be seen strictly as a tree of one gene only: it is not a tree of life. We cannot infer what a cell might have looked like, or how it might have lived in the past, on the basis of its ribosomal genotype. Eukaryotes are now plainly seen to be genomic chimeras, apparently formed in a singular endosymbiosis between an archaeal host cell and a bacterium around 1.5 billion years ago [50]. This chimerism cannot be depicted on a normal branching phylogenetic tree, because endosymbiosis involves fusion of branches, not bifurcation, producing instead a striking composite tree, depicted beautifully (and presciently, as this is still accurate) by Bill Martin in 1998 [51] (figure 5). Bill Martin and I have since argued that the singular endosymbiosis at the origin of eukaryotes, which gave rise to mitochondria, increased the energy available per gene in eukaryotic cells by a breath-taking three to five orders of magnitude [52]. That overcame the pervasive energetic constraints faced by bacteria, enabling a massive expansion in cell volume and genome size, and permitting the evolution of many eukaryotic traits from the nucleus to sex and phagocytosis (all of which were first reported by Leeuwenhoek himself). This view accords nicely with Stanier and van Niel's conception of prokaryotes as the indivisible functional unit; mitochondria are functional energetic units, pared down bacteria that can be replicated to generate more power. It might be that eukaryotes *had to* evolve by way of an endosymbiosis, for these bioenergetic reasons.

**Figure 5.**
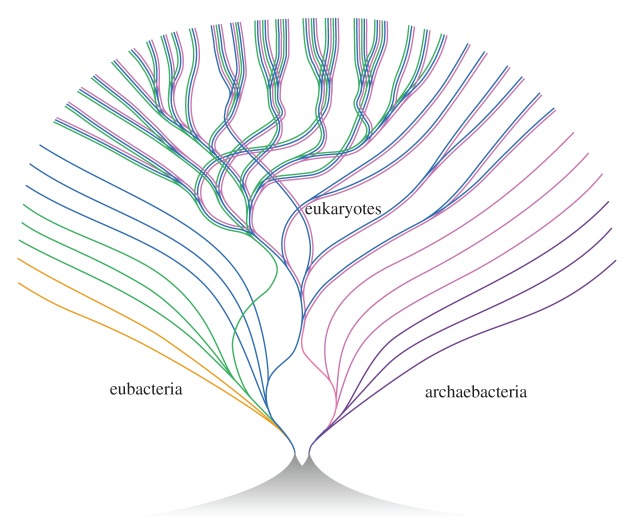
A tree of life drawn by Bill Martin in 1998, reflecting whole genomes. The tree shows the chimeric origin of eukaryotes, in which an archaeal host cell acquired bacterial endosymbionts that evolved into mitochondria; and the later acquisition of chloroplasts in *Plantae*. Reproduced with permission from [51]. Copyright 1999 © John Wiley & Sons, Inc.

Even in the absence of endosymbiosis, the idea of a true phylogenetic tree of life is undermined by the prevalence of lateral gene transfer in both bacteria and archaea. Informational genes, including ribosomal RNA, are generally inherited vertically, giving a robust phylogenetic signal, but such genes account for barely 1% of a bacterial genome, and much of the rest is passed around between cells by lateral gene transfer, confounding deep phylogenetic signals. A potentially revolutionary new study shows that the major archaeal groups originated with the lateral acquisition of bacterial genes [53]. Ironically, the unity of biochemistry—Kluyver's edifying guide to evolution—is the root problem: the universality of the genetic code, intermediary metabolism and energy conservation (e.g. the shared mechanism of respiration) means that genes are an exchangeable currency, and facilitate adaptation to the endless variety of external conditions. Again, the link between the ribosomal genotype of a prokaryotic cell and its phenotype—the way it makes a living—is forever changing. Ford Doolittle notes that pervasive genetic chimerism means that ‘no hierarchical universal classification can be taken as natural’ [54]; the universal tree of life is a human foible and not a true representation of the real world. As Doolittle observes, ‘Biologists might rejoice in and explore, rather than regret or attempt to dismiss, the creative evolutionary role of lateral gene transfer’ [54]. The tree of life promises a hierarchical order, and takes authority from Darwin himself, but in microbes at least it is not sustained by the very genetic sequences that made such phylogeny possible. ‘Early evolution without a tree of life’ [55] might seem an alarming vista to many, but Leeuwenhoek would surely have felt at home. He was happiest without a compass.

Perhaps that, more than anything else, is the lesson we still need to learn from Leeuwenhoek today. There is a danger of complacency in biology, a feeling that the immense computational power of the modern age will ultimately resolve the questions of biology, and medical research more broadly. But pathophysiology stems from physiology, and physiology is a product of evolution, largely at the level of cells. The eukaryotic cell seems to have arisen in a singular endosymbiosis between prokaryotes, and eukaryotes share a large number of basic traits, few of which are known in anything like the same form in bacteria or archaea. We know of no surviving evolutionary intermediates between prokaryotes and eukaryotes. We know almost nothing about which factors drove the evolution of many basal eukaryotic traits, from the nucleus to meiosis and sex, to cell death—traits first observed by Leeuwenhoek. Why did meiosis and sex arise from lateral gene transfer in bacteria? Why did the nucleus evolve in eukaryotes but not in bacteria or archaea? What prevents bacteria from engulfing other cells by phagocytosis? There is no agreement on the answers to these questions, nor more broadly to a question that might easily have been asked by Leeuwenhoek himself—why is life the way it is? Some of us have argued that eukaryotic evolution is explicable in terms of the detailed mechanisms of energy conservation, with an allied requirement for endosymbiosis leading to conflict and coadaptation between endosymbionts and their host cells [56]. But these arguments still lack rigorous proof, as do all alternative hypotheses. In the meantime, we have at best an unreliable map of the land that enchanted Leeuwenhoek. We should rejoice and explore.

## Author profile

**Nick Lane** is a Reader in Evolutionary Biochemistry in the Department of Genetics, Evolution and Environment at University College London. His research is on the role of bioenergetics in the origin of life and the early evolution of cells, focusing on the importance of endosymbiosis and cellular structure in determining the course of evolution. Nick was awarded the inaugural UCL Provost's Venture Research Fellowship in 2009, the BMC Research Award for Genetics, Genomics, Bioinformatics and Evolution in 2011, and the Biochemical Society Award for 2015. He leads the UCL Research Frontiers Origins of Life programme and was a founding member of the UCL Consortium for Mitochondrial Research. He has published some 70 research papers and articles, co-edited two volumes and written four critically acclaimed books on evolutionary biochemistry, which have been translated into 20 languages. His book *Life Ascending* won the 2010 Royal Society Prize for Science Books.
